# Discovery of Species-unique Peptide Biomarkers of Bacterial Pathogens by Tandem Mass Spectrometry-based Proteotyping*[Fn FN2]

**DOI:** 10.1074/mcp.RA119.001667

**Published:** 2020-01-15

**Authors:** Roger Karlsson, Annika Thorsell, Margarita Gomila, Francisco Salvà-Serra, Hedvig E. Jakobsson, Lucia Gonzales-Siles, Daniel Jaén-Luchoro, Susann Skovbjerg, Johannes Fuchs, Anders Karlsson, Fredrik Boulund, Anna Johnning, Erik Kristiansson, Edward R. B. Moore

**Affiliations:** ‡ Department of Infectious Diseases, Institute of Biomedicine, Sahlgrenska Academy of the University of Gothenburg, SE-40234 Gothenburg, Sweden; § Department of Clinical Microbiology, Sahlgrenska University Hospital, SE-413 46 Gothenburg, Region Västra Götaland, Sweden; ¶ Culture Collection University of Gothenburg (CCUG), Sahlgrenska Academy of the University of Gothenburg, SE-41346 Gothenburg, Sweden; ‖ Centre for Antibiotic Resistance Research (CARe), University of Gothenburg, SE-40234 Gothenburg, Sweden; ** Proteomics Core Facility at Sahlgrenska Academy, University of Gothenburg, SE- 40530 Gothenburg, Sweden; ‡‡ Nanoxis Consulting AB, SE-40016 Gothenburg, Sweden; §§ Microbiology, Department of Biology, University of the Balearic Islands, E-07122, Palma de Mallorca, Spain; ¶¶ Center for Translational Microbiome Research (CTMR), Department of Microbiology, Tumor and Cell Biology, Karolinska Institute, Stockholm, Sweden; ‖‖ Department of Mathematical Sciences, Chalmers University of Technology, SE-41296 Gothenburg, Sweden; ‡‡‡ Department of Systems and Data Analysis, Fraunhofer-Chalmers Centre, Chalmers Science Park, SE-412 88 Gothenburg, Sweden

**Keywords:** Biomarker: diagnostic, bacteria, early diagnosis, tandem mass spectrometry, infectious disease, microbiology, peptides

## Abstract

Mass spectrometry (MS) and proteomics offer comprehensive characterization and identification of microorganisms and discovery of protein biomarkers that are applicable for diagnostics of infectious diseases. The use of biomarkers for diagnostics is widely applied in the clinic and the use of peptide biomarkers is increasingly being investigated for applications in the clinical laboratory. Respiratory-tract infections are a predominant cause for medical treatment, although, clinical assessments and standard clinical laboratory protocols are time-consuming and often inadequate for reliable diagnoses. Novel methods, preferably applied directly to clinical samples, excluding cultivation steps, are needed to improve diagnostics of infectious diseases, provide adequate treatment and reduce the use of antibiotics and associated development of antibiotic resistance. This study applied nano-liquid chromatography (LC) coupled with tandem MS, with a bioinformatics pipeline and an in-house database of curated high-quality reference genome sequences to identify species-unique peptides as potential biomarkers for four bacterial pathogens commonly found in respiratory tract infections (RTIs): *Staphylococcus aureus*; *Moraxella catarrhalis*; *Haemophilus influenzae* and *Streptococcus pneumoniae*. The species-unique peptides were initially identified in pure cultures of bacterial reference strains, reflecting the genomic variation in the four species and, furthermore, in clinical respiratory tract samples, without prior cultivation, elucidating proteins expressed in clinical conditions of infection. For each of the four bacterial pathogens, the peptide biomarker candidates most predominantly found in clinical samples, are presented. Data are available via ProteomeXchange with identifier PXD014522. As proof-of-principle, the most promising species-unique peptides were applied in targeted tandem MS-analyses of clinical samples and their relevance for identifications of the pathogens, *i.e.* proteotyping, was validated, thus demonstrating their potential as peptide biomarker candidates for diagnostics of infectious diseases.

## Abstract (precis)

Peptide biomarker candidates for the respiratory tract pathogens *Streptococcus pneumoniae*, *Haemophilus influenzae*, *Moraxella catarrhalis* and *Staphylococcus aureus* are presented. First, bacterial cultures representing the genetic variability in each of the four species, were analyzed. The peptide biomarker candidates were then experimentally verified to be present in a clinical situation by analyzing true positive clinical samples. The most promising peptide biomarkers were used in a targeted MS mode, demonstrating their use for future clinical implementation.

## Graphical Abstract

**Figure:**
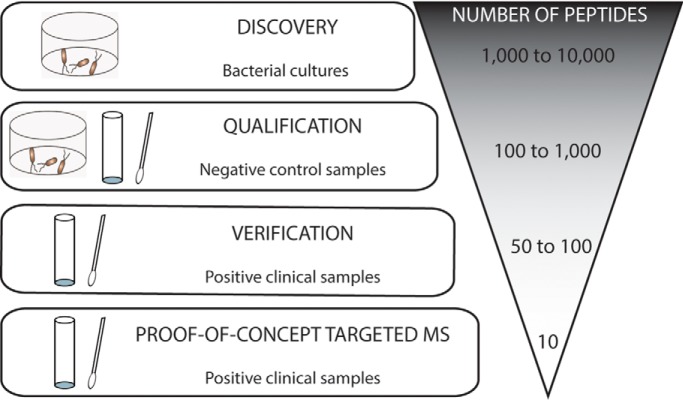

**Highlights**

- Discovery of peptide biomarker candidates of respiratory tract pathogens *S. pneumoniae*, *H. influenzae*, *M. catarrhalis* and *S. aureus* as target pathogens.
- Peptide biomarker candidates were experimentally verified in clinical samples.
- Targeted MS using promising peptide biomarker candidates shown as proof-of-concept.

Respiratory tract infections (RTIs)^1^ are a major reason for hospital admissions and are often treated with antibiotics (1). Today, a clinical assessment performed by the physician, is mainly based on symptoms, together with supporting clinical laboratory microbiological confirmation (2). Microbiological characterization of a clinical sample traditionally relies on cultivation of bacteria, which not only takes precious time, but in many cases is inconclusive because of the difficulty to recover viable bacteria. For example, in only ∼50% of the cases, are *Streptococcus pneumoniae*, a responsible agent for pneumococcal infections, recovered by culturing (3). Because bacterial infection can lead rapidly to invasive life-threatening situations, physicians may prescribe broad-spectrum antibiotics before knowing whether the infection is caused by bacteria or virus. Overuse of broad-spectrum antibiotics is a significant contributor to the emergence of anti-microbial resistance (AMR). One of the key counter-measures in the battle against AMR will be the development of improved, rapid, accurate and comprehensive diagnostic methods.

DNA-based diagnostic approaches, such as real-time polymerase chain reaction (RT-PCR) is currently implemented in the routine protocols of the clinical microbiology laboratory and whole-genome sequencing is increasingly applied. However, PCR is a targeted approach and, thus, detects and identifies only the known and selected targets, which can lead to biased results and insufficient species resolution and characterization. One example is in the differentiation of closely related species within the Mitis Group of the genus *Streptococcus*, using PCR-based analyses of house-keeping genes or virulence factors (4–6).

Matrix-Assisted Laser Desorption/Ionization-Time-Of-Flight (MALDI-TOF) MS-based microbial species identification has emerged as an alternative to traditional phenotypic- or genotypic-based methods (7–10). Demonstrating benefits, such as reliable species-level resolution, in most cases, ease-of-use and speed of processing samples, as well as low cost per analysis, MALDI-TOF MS identification is now used in clinics world-wide. However, a significant drawback of MALDI-TOF MS analyses is that it, in most cases, requires time-consuming cultivation and isolation of the relevant microorganisms. Further drawbacks include limitations in discriminating closely related species, including some species of the Mitis Group of the genus *Streptococcus*, and, except in some limited cases (11–13), it has proven ineffective for obtaining information on characteristic features, such as AMR and virulence (14).

To increase the discriminative power and resolution for differentiating closely related species, even to strain-level typing, tandem MS approaches at the peptide level have been employed (14–21). Peptide biomarker discovery has been facilitated by development of MS-instruments performing bottom-up “high-resolution accurate-mass (HRAM)” tandem MS proteomics, enabling identification of thousands of peptides simultaneously, in a single analysis (16). At the peptide level, tandem MS has the power to elucidate expressed point mutations (22), enabling high levels of resolution. Biomarkers for resistance and virulence factors can be detected simultaneously in the same analysis, providing crucial information for diagnoses and proper treatments (23, 24).

Previously, we have shown that peptide biomarkers have the power to differentiate bacterial species (14), as well as strains within the same species (18). This “proteotyping” approach (14, 25, 26) can also be used for differentiating taxonomically-close species, such as the pathogen *S. pneumoniae* from commensal species, *S. pseudopneumoniae* and *S. miti*s of the Mitis Group of the genus *Streptococcus* (14). In the present study, the workflow combines HRAM tandem MS and the TCUP (Typing and Characterization of bacteria Using bottom-up tandem mass spectrometry Proteomics) bioinformatics pipeline (27) in the search for novel species-unique peptides as potential biomarkers for the respiratory tract pathogens, *Staphylococcus aureus, Moraxella catarrhalis, Haemophilus influenzae* and *Streptococcus pneumoniae*. In contrast to traditional cultivation-based methodologies, proteotyping is not relying on recovery of cultivable cells, but can be applied directly to clinical samples. The purpose of this study was to initially identify species-unique peptides as potential peptide biomarker candidates from bacterial cultures of reference strains of the target bacterial species and then to confirm these biomarker candidates in clinical respiratory-tract samples without any cultivation step (Fig. 1).

## EXPERIMENTAL PROCEDURES

### 

#### 

##### Cultivation and Classification of Bacteria

Bacterial strains were selected of each of four common respiratory-tract infectious bacterial species: *S. aureus* (12 strains), *M. catarrhalis* (11 strains), *H. influenzae* (9 strains), and *S. pneumoniae* (7 strains); obtained from the Culture Collection, University of Gothenburg, Gothenburg, Sweden (CCUG; www.ccug.se) (supplemental Table S1). Cultures were grown overnight in the following way: *S. aureus* was grown on Blood Agar, at 37 °C, aerobically; *M. catarrhalis* and *S. pneumoniae* were grown on Blood Agar, at 37 °C, with 5% CO_2_; *H. influenzae* strains were grown on Chocolate Agar medium, at 36 °C, with 5% CO_2_. The classifications of the selected strains of *H. influenzae*, *M. catarrhalis* were confirmed by 16S rRNA gene sequence determinations and comparative sequence analyses (28). Classifications of the selected strains of *S. aureus* were confirmed by 16S rRNA gene and *sodA* sequence analyses (29). Classifications of the selected strains of *S. pneumoniae* were confirmed by whole genome sequence Average Nucleotide Identity based on BLAST (ANIb) analyses (30), using JSpeciesWS (31), against the genome sequence of *S. pneumoniae* NCTC 7465^T^ (GenBank accession number: LN831051).

##### Peptide Generation from Bacterial Cultures

Bacterial biomass was collected from fresh cultures and suspended in phosphate-buffered saline (PBS). The bacteria were washed with PBS and lysed, by bead beating (14). The bacterial lysates were frozen until further analysis. The Lipid-based Protein Immobilization (LPI^®^) methodology was employed for generating peptides from the cultured bacteria, as described previously (14, 18, 27). Each strain of the four bacteria, *S. aureus, M. catarrhalis, H. influenzae,* and *S. pneumoniae,* were digested in triplicates (supplemental Fig. S1).

To digest bacterial proteins into peptides, the cell lysate was injected into a LPI Hexalane FlowCell (Nanoxis Consulting AB, Gothenburg, Sweden, www.nanoxisconsulting.com; Patent Application No. WO2006068619), using a pipette to fill the FlowCell channel (channel volume of ∼30 μl). Proteins were immobilized to the FlowCell surface, after incubation for 1 h, at room temperature. The FlowCell channels were washed with 400 μl of ammonium bicarbonate, using a syringe pump, with a flow rate of 100 μl/min. Enzymatic digestion of the proteins was performed by injecting trypsin (V5111, Promega, Madison, WI) (2 μg/ml in 20 mm ammonium bicarbonate, pH 8.0) into the FlowCell channels and incubating for 1 h at room temperature. The generated peptides were eluted by injecting 200 μl ammonium bicarbonate buffer (20 mm, pH 8.0) into the channels. The eluted peptides were collected at the outlet ports, using a pipette, and transferred into tubes (2.0 ml, Axygen, Corning Life Sciences, MA). The peptide solutions were incubated at room temperature overnight and subsequently frozen at −20 °C until analysis by MS. The peptide samples were not reduced or alkylated prior to MS analysis.

##### Clinical Samples

Clinical respiratory tract samples (nasopharyngeal and nasal swabs, *n* = 218), analyzed and reported as positive by the Clinical Microbiology Laboratory (Sahlgrenska University Hospital, Gothenburg, Sweden), were collected in Amies media (eSwab, Copan Diagnostics, Inc, CA). The clinical samples were reported to contain at least one of the four pathogens included in the study (*S. aureus, M. catarrhalis, H. influenzae* and/or *S. pneumoniae*). In many cases, the samples displayed co-infection with two or more of these pathogens. The pathogens in clinical samples were confirmed by the standard, accredited clinical microbiology laboratory protocols for selective and differential isolation of bacteria, including subsequent identification by MALDI-TOF MS analysis. Samples were supplemented with STGG (Skim milk, Tryptone, Glucose, Glycerol) to bolster the viability of bacteria as well as recovery of bacterial proteins during storage of respiratory tract samples and frozen until processing (32). Only samples that were collected as part of the standard diagnostic protocols were included in this study; no additional or extra sampling from patients was carried out and no patient identifiable information was collected; hence, informed consent was not required.

In the qualification phase, clinical respiratory tract samples, reported to be negative for bacteria by cultivation-based protocols and MALDI-TOF-MS, were spiked with cells of the type strains of the four species *H. influenzae* (CCUG 23945^T^), *M. catarrhalis* (CCUG 353^T^), *S. aureus* (CCUG 41582^T^) and *S. pneumoniae* (CCUG 28588^T^), to select the most promising peptide biomarker candidates for the validation phase. The number of added cells to the negative clinical samples ranged from 100 cells/ml to 1 million cells/ml (supplemental Fig. S2).

##### Peptide Generation from Clinical Samples

The MolYsis kit (MolYsis Basic5 kit, Molzym GmbH & Co. Bremen, Germany) was used for removal of human biomass, according to the supplier's protocol, with minor modifications. After sample treatment, the resulting bacterial pellets were re-suspended in 120 μl ammonium bicarbonate (20 mm pH 8) and bacteria were lysed, using bead beating (14). For digestion of proteins, to generate peptides, sodium deoxycholate (SDC, 5% in 20 mm ammonium bicarbonate, pH 8) was added to 1% (w/v) final concentration. Trypsin (2 μg/ml, 100 μl ammonium bicarbonate, 20 mm pH 8) was added and samples were digested for ∼8 h at 37 °C. SDC was removed by precipitation by addition of formic acid (FA) followed by centrifugation at 13,000 × *g* for 10 min. Supernatants containing the peptides were stored at −20 °C until analysis. The peptide samples were not reduced or alkylated prior to MS analysis (supplemental Fig. S3).

##### NanoLC-MS/MS Analysis

Peptide samples were desalted, using PepClean C18 spin columns (Thermo Fisher Scientific, MA), according to the manufacturer's guidelines. MS analyses were carried out, using Q Exactive or a QExactive HF MS (Thermo Fisher Scientific) interfaced with an Easy nLC 1200 liquid chromatography system (Thermo Fisher Scientific). Peptides were trapped on an Acclaim Pepmap 100 C18 trap column (100 μm × 2 cm, particle size 5 μm, Thermo Fischer Scientific) and separated on an in-house packed analytical column (75 μm × 300 mm, particle size 3 μm, Reprosil-Pur C18, Dr. Maisch, Germany), using a gradient from 7% to 35% B over 35, 50, or 75 min followed by an increase to 100% B for 5 min at a flow of 300 nL/min. Solvent A was 0.2% formic acid and solvent B was 80% acetonitrile in 0.2% formic acid. The instrument operated in data-dependent mode where the precursor ion mass spectra were acquired at a resolution of 70,000 (QE) or 60,000 (QEHF), the 10 most intense multiply charged ions were isolated in a 2.0 Da isolation window and fragmented using collision energy HCD settings at 27. MS2 spectra were recorded at a resolution of 35,000 (QE) or 30,000 (QEHF). Dynamic exclusion was set to 20–30 s with 10 ppm tolerance. Inclusion lists, containing the candidate peptide biomarkers for each species, were used in the qualification and verification phases together with pick others to improve the sensitivity of the MS-method. The *m/z* ratios (a maximum of 50 for each MS-analysis) corresponding to specific peptide biomarkers were prioritized for fragmentation even if they were not among the Top10 most abundant peptides.

##### TCUP Bioinformatics Pipeline

Raw data were evaluated using the TCUP bioinformatics pipeline (27) to identify species-unique peptides. The LC-MS/MS output was converted from the proprietary Thermo Xcalibur RAW format to the open-source mzXML format (33), using ReAdW (34) (version 201411.xcalibur), with command-line arguments: “–nocompress -gzip.” The X! Tandem spectrum search engine (version VENGEANCE Dec. 15, 2015) (35, 36) was used to identify peptides from the mass spectra with the following settings: fragment monoisotopic mass error = 20; parent monoisotopic mass error plus = 5; parent monoisotopic mass error minus = 5; fragment mass type monoisotopic, dynamic range = 100.0; total peaks = 50; maximum parent charge = 4; minimum parent m+h = 800.0; minimum fragment *m*/*z* = 100.0, minimum peaks = 15, potential modification mass = 16.0@M, maximum valid expectation value = 1.0. In addition, X! Tandem peptides were also filtered to only allow peptides with a hyperscore of >30 in downstream analyses (37). Values for all X!Tandem settings are available in supplemental file 1. The reference database used in this step was a customized database consisting of 56,967,781 non-redundant proteins from the NCBI GenBankTM NR (38) and 6,320,906 peptide sequences from the reference genomes archived within the Human Microbiome Project (39). All sequences containing unidentified peptides (“X”), as well as duplicates of sequences shared between the two databases, were removed. The resulting database used with X! Tandem contained a total of 59,349,300 distinct protein sequences. The taxonomic hierarchy used in TCUP was based on the complete NCBI Taxonomy (40) (taxdump downloaded Nov. 17, 2015) and each reference genome in the reference database was associated with a unique node in the taxonomic tree. The search parameters were set, according to Boulund *et al.* (27). All peptides presented in Tables I–IV were mapped against RefSeq sequences (Oct 2018) using BLAST (https://blast.ncbi.nlm.nih.gov/Blast.cgi).

##### Generation of Targeted Database and MS-inclusion Lists

A targeted database was compiled, including 15,417 species-unique peptides identified by TCUP in at least one of the MS analyses of the representative strains of the four bacterial species (Table V, supplemental Table S2). The smaller targeted database was used for increasing the probability of positive identification of the relevant peptide biomarkers. Additionally, MS-inclusion lists used in the later qualifications phase were generated. Peptides that were detected in all strains and all MS analyses were ranked the highest in the lists (supplemental Fig. S1). The lists contain the 100 highest-ranked peptides for each species.

These peptide lists were revised after the qualification phase and the peptides detected in the samples with a lower number of spiked pathogenic bacteria were ranked the highest. In verification phase, the clinical samples were analyzed in batches and the peptide lists were again revised after each batch, according to the following criteria: (1) Identified peptides from the list were verified as a peptide biomarker candidate by its presence in clinical samples; (2) Peptides identified in the clinical samples by TCUP, but not present in the inclusion lists, were added to updated versions of the inclusion lists; (3) Peptide biomarker candidates present in the initial inclusion lists, but not detected in the clinical samples, were removed from updated inclusion lists or were given a lower ranking (supplemental Data S1–S4). After ranking, the final lists of peptides for each of the bacterial species were reduced to the top 16–18 peptide biomarker candidates (supplemental Fig. S3, Tables I–IV).

##### Database Matching

In parallel with the TCUP bioinformatics pipeline, the data was matched, using Proteome Discoverer (Thermo Fisher Scientific, version 1.4), against the targeted database. Mascot 2.5 (Matrix Science, MA) was used as a search engine with precursor mass tolerance of 5 ppm and fragment mass tolerance of 200 mmu and variable methionine oxidation. Fixed Value with a maximum delta Cn of 0.05 was employed in the database matching and the peptides used for protein identification were filtered at 1% FDR. The fragmentation spectra and ion series for all detected peptides in the clinical samples were inspected manually to verify correct identifications.

##### Targeted MS (PRM) Analyses

For each of the four bacterial species, the top 16–18 peptide biomarker candidates (Tables I–IV, supplemental Data S5–S8), most prominently found in clinical samples were analyzed, using parallel reaction monitoring (PRM) on a Q Exactive HF (Thermo Fisher Scientific). Separation was performed, using a 50 min gradient, as stated above and the precursor ions of the peptides were targeted without scheduling. The QEHF orbitrap resolution was 30 000, a quadrupole isolation window of 1.2 Da and collision energy HCD settings at 27 were used. PRM data were analyzed using Skyline (version 4.2.0) (39). Peak picking was manually checked and corrected in accordance with the retention time, transitions and mass accuracy to confirm the identities of peptides. For this proof-of-concept, the PRM method used here was employed to bias toward detection of the peptide biomarkers of interest (Tier 3, as defined in the MCP guidelines).

The MS proteomics data (MS/MS-spectra for all species-unique peptides presented in Tables I–IV, as well as raw-files and PD1.4 search files of representative clinical samples) have been deposited to the ProteomeXchange Consortium via the PRIDE partner repository with the data set identifiers PXD014522.

##### Experimental Design and Statistical Rationale

**Fig. 1.**
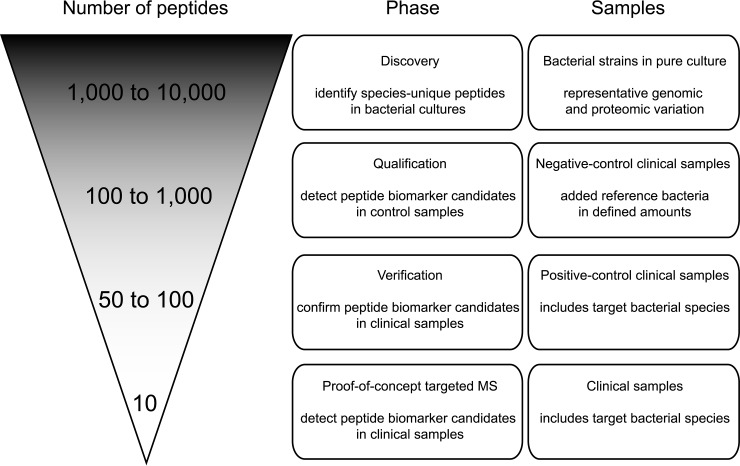
**Illustration showing the process employed for identifying species-unique peptides as potential peptide biomarker candidates.** During the process, bacterial cultures, representing genomic and proteomic variation within the species, as well as clinical samples, were analyzed. The purpose of this workflow was to initially identify as many species-unique peptides as possible and in later phases narrow down the number of peptides to the most promising peptide biomarker candidates to be used for diagnostic analyses.

The workflow of how to discover, qualify and verify the species-unique peptides is shown in Fig. 1 and supplemental Fig. S4. The number of bacterial strains included in the discovery phase of finding species-unique peptides were *S. aureus* (12 strains), *M. catarrhalis* (11 strains), *H. influenzae* (9 strains) and *S. pneumoniae* (7 strains), all analyzed in triplicate, resulting in, at least, 21 MS analyses per species (Table V). The number of identified species-unique peptides increased with the number of analyzed strains. However, at a certain stage, analyses of additional strains did not contribute to further increase in the number of species-unique peptides and the number of strains selected per species was concluded to be satisfactory (supplemental Figs. S5–S8).

In the verification phase, to verify the presence of the species-unique peptides in patient samples, without prior culturing, the number of clinical samples included was 218. As this study was focused on the discovery of species-unique peptides no replicate analyses were performed at this stage, as it was deemed more important to analyze many individual clinical samples.

## RESULTS

In summary, the workflow of discovering, qualifying and verifying the species-unique peptides as promising peptide biomarker candidates was divided into four phases (Fig. 1 and supplemental Fig. S4). First, in the discovery phase, species-unique peptides were identified from pure bacterial cultures. Subsequently, in the qualification phase, negative clinical samples were spiked with bacterial cells in order to ensure that the peptides could be detected in the context of a realistic clinical sample. In the verification phase, positive clinical samples were analyzed to verify the species-unique peptides most frequently found in clinical samples. Last, as a proof-of-concept, positive clinical samples were analyzed using a targeted MS approach with the selected candidate peptide biomarkers as targets.

In the discovery phase, several representative strains from each of the four target species, *S. aureus* (13 strains), *M. catarrhalis* (11 strains), *H. influenzae* (9 strains) and *S. pneumoniae* (7 strains) were selected to reflect the genetic variation within the species (supplemental Table S1). Each species was analyzed with a minimum of 21 MS runs resulting in identified species-unique peptides (Table V). The largest number of species-unique peptides were found in *S. aureus* and *M. catarrhalis* (5847 and 5810, respectively), *H. influenzae* strains comprised 2978 species-unique peptides, whereas the fewest number of species-unique peptides (782) was detected in strains of *S. pneumoniae*. The peptides were ranked, based on the number of strains in which they were detected. These results from the MS-analyses were compiled to a database containing the 15,417 species-unique peptides (supplemental Table S2). The most promising peptide biomarker candidates, based on the number of strains they were found in, were reduced to lists of 100 peptides for each species (supplemental Fig. 1).

In the qualification phase, the suitability of the species-unique peptides as potential peptide biomarker candidates was evaluated. Negative clinical samples were spiked with varying concentrations of bacterial cells (supplemental Fig. S2) and the MS analyses were performed using inclusion lists with the hundred highest ranking species-unique peptides identified in the discovery phase. The number of bacterial cells per ml of sample ranged from 100 to 1 million cells/ml, reflecting the variation in the number of bacteria cells typical for nasopharyngeal/nasal swab samples; bacterial loads vary during different phases of infection and are also dependent on the pathogen (41, 42). The selected range was considered to realistically reflect both weakly- and strongly-infected samples. The species-unique peptides detected in samples containing the lowest number of bacterial cells, ranging from 1000 to 10,000, were deemed to be promising peptide biomarker candidates (supplemental Table S3). The ranking of the peptides in the respective inclusion lists were revised in accordance with the results from the qualification phase (supplemental Fig. S2).

In the verification phase, 218 clinical respiratory tract samples (312 MS injections) were included, from which isolations of *S. aureus, M. catarrhalis, H. influenzae* and/or *S. pneumoniae*, were reported. These samples were thus used as “positive control” samples for MS analyses. The clinical samples were reported by the Clinical Microbiology Laboratory to contain at least one of the pathogens included in the study, but in many cases, samples displaying co-infection with two or more of these four pathogens, were included. By analyzing these samples, peptide biomarker candidates were detected and identified, confirming that the proteins from which the peptide biomarker candidates originate, are present *in vivo*. The peptides most prominently detected in clinical samples, and their corresponding proteins, as well as the number of times they were detected in the cultures of bacterial reference strains, are presented in Tables I, II, III, and IV. In order to verify the identities of the peptides in the clinical samples, all fragmentation spectra were inspected manually. The fragmentation spectra and ion series for a top ranked peptide for each of the four species are shown in supplemental Figs. S9–S12. During the analysis of the clinical samples in the verification phase, the lists containing the peptide biomarker candidates were continuously revised according to the ranking of the peptide. The final lists were reduced to contain only the 15–20 most promising peptide biomarker candidates for the proof-of-concept targeted MS analyses (supplemental Fig. 3, supplemental Data S1–S4).

**Table I:** The peptide biomarker candidates of S. aureus and the proteins from which they originate

Peptide sequence	Number of times detected in 36 MS analyses of *S. aureus* cultures	Number of times detected in unique clinical samples	Protein (GenBank accession number and description)	
TVQPIDVDTIVASVEK	36	22	AKJ16950.1	2-oxoisovalerate dehydrogenase
QAGVGAAVVAELSER	36	18		
ELINNIQSGQR	36	15	AKJ17520.1	Preprotein translocase subunit YajC
LGISDGDVEETEDAPK	36	16	AKJ17148.1	Recombinase RecA
ALLNNMVQGVSQGYVK	36	14	AKJ18065.1	50S ribosomal protein L6
SNVNDATDYSSETPEGK	36	12	AKJ17216.1	Transketolase
ANNVATDANHSYTSR	36	13	AKJ17623.1	Hypothetical protein
ILAESPNLAISSSSR	35	10	AKJ16422.1	HAD family hydrolase
NVVEIPLNDEEQSK	31	9	AKJ16109.1	Lactate dehydrogenase
ATEATNATNNQSTQVSQATSQPINFQVQK	24	7	AKJ16987.1	Heme transporter IsdA
IHLVGDEIANGQGIGR	35	8	AKJ17576.1	Pyruvate kinase
NISNNVLVTIDAAQGK	13	6		
TAKPVAEVESQTEVTE	26	10	AKJ16406.1	DNA-directed RNA polymerase subunit beta'
SQGVSEEELNESIDR	29	1	AKJ16022.1	Acetaldehyde dehydrogenase
AEENGLTVVDAFNFEAPK	16	7	AKJ18079.1	50S ribosomal protein L4
LLGINATIVMPETAPQAK	1	1	AKJ17317.1	Threonine dehydratase

**Table II:** The peptide biomarker candidates of M. catarrhalis and the proteins from which they originate

Peptide sequence	Number of times detected in number of 33 MS analyses of *M. catarrhalis* cultures	Number of times detected in unique clinical samples	Protein (GenBank accession number and description)	
VVLAGDTVVSDR	33	14	WP_003666427.1	TonB-dependent receptor
QIVSNAGDEASVIVNEVK*	33	18	WP_063454121.1	Chaperonin GroEL
AIAQVGSISANSDATIGELISK	29	16		
ELSNTAAETQPK	33	18	WP_003659702.1	30S ribosomal protein S1
VDATVDAQNPTK	24	16	WP_003660336.1	Hypothetical protein
QSDVGQLTGK	5	9		
FNATAALGGYGSK	31	12	WP_063454085.1	Cell surface protein
THTSALAEENQQASIPR	33	12	WP_063454087.1	Cell division protein FtsZ
YVVEGANMPLDAQAIDIVR	17	11	WP_049156084.1	NADP-specific glutamate dehydrogenase
SQIYQTTASVSGAR	33	9	WP_003657351.1	Ohr family peroxiredoxin
LLNETTGQVVPK	33	8	WP_003657987.1	DUF4377 domain-containing protein
SSENVVVVSVR	33	10	WP_063454071.1	Electron transfer flavoprotein subunit beta
AISYGNSADAQPYVGAK	33	10	WP_003658939.1	Porin family protein
GLPVSNSGAPISVPVGQATLGR	31	8	WP_003658974.1	F0F1 ATP synthase subunit beta
VNYNGDTDTVTLSGVAK	33	13	WP_003656943.1	Peptidoglycan-binding protein LysM
AVATQQATVSAEYLQK	5	10	WP_003657125.1	ABC transporter substrate-binding protein
ADSGLSESEIEEMIR	32	12	WP_003669031.1	Molecular chaperone DnaK
LGAQEAELVSNSK	33	7	WP_003660298.1	CTP synthase

*This peptide was also found in samples spiked with the fewest number of cells (supplemental Table S2).

**Table III:** The peptide biomarker candidates of H. influenzae and the proteins from which they originate

Peptide sequence	Number of times detected 26 MS analyses of *H. influenzae* cultures	Number of times detected in unique clinical samples	Protein (GenBank accession number and description)	
GVAADAISATGYGK*	22	22	WP_038441355.1	Porin OmpA
ANLKPQAQATLDSIYGEMSQVK	5	6		
ADSVANYFVAK	−	5		
GSYEVLDGLDVYGK	12	3		
LSQERADSVANYFVAK**	−	2		
AVVYNNEGTNVELGGR*	22	14	WP_058222193.1	Porin
YDANNIIAGIAYGR*	13	6		
ATHNFGDGFYAQGYLETR	15	5		
AVVYNNEGTKVELGGR	−	5		
QQVNGALSTLGYR	18	1		
YVPTNGNTVGYTFK	−	4		
LSVIAEQSNSTR*	4	1		
SADLTNEVAVGDVVEAK	4	6	WP_011272719.1	30S ribosomal protein S1
SADLTSEVAVGDVVEAK	11	2		
TSPTQNLSLDAFVAR	9	5	WP_058222202.1 WP_050846043.1	ShlB/FhaC/HecB family hemolysin secretion/activation protein
AQYIVEQVIGQAR	26/29	2	WP_011272712.1	Pyruvate dehydrogenase (acetyl-transferring), homodimeric type

*This peptide was also found in samples spiked with the fewest number of cells (supplemental Table S2).

**Peptide with missed cleavage includes ADSVANYFVAK.

**Table IV:** The peptide biomarker candidates of S. pneumoniae and the proteins from which they originate

Peptide sequence	Number of times detected in 21 MS analyses of *S. pneumoniae* cultures	Number of times detected in unique clinical samples	Protein (GenBank accession number and description)	
VSDVAESTGEFTSEQFEK*	21	22	WP_000064115.1	Asp23/Gls24 family envelope stress response protein
GAANGVVSHENTR*	−	9		
EEAPVASQSK	−	9	WP_001035310.1	Hypothetical protein
SADQQAEEDYAR	−	8		
APLQSELDTK	−	3		
LKEIDESDSEDYVK	−	3		
NVEIIEDDKQGVIR	1	10	WP_000245505.1	30S ribosomal protein S8
NLPVGSDGTFTPEDYVGR	20	8	WP_001291372.1	Methionine–tRNA ligase
TLELEIAESDVK	−	5	WP_000458177.1	Hypothetical protein
DIGLANDGSIVGINYAK	12	5	WP_000927809.1	Sugar ABC transporter substrate-binding protein
IAELEYEVQR	−	6	WP_001008677.1	Asp-tRNA(Asn)/Glu-tRNA(Gln) amidotransferase subunit GatB
AVAAADAADAGAAK	3	3	WP_001196960.1	50S ribosomal protein L7/L12
GQDWVIAAEVVTKPEVK	16	5	WP_000116461.1	Trigger factor
TLSPEEYAVTQENQTER	−	6	WP_000998307.1	Peptide-methionine (R)-S-oxide reductase
KDEAEAAFATIR	−	3	WP_001284361.1	Thiol-activated toxin pneumolysin
SQPSSETELSGNKQEQER	16	2	WP_078148305.1	Sialidase
IGVISVVEDGDEALAK	−	2	WP_000808063.1	Elongation factor Ts
VAYFNEIDTYSEVK	−	2	WP_000685088.1	Nucleotide sugar dehydrogenase

*This peptide was also found in samples spiked with the fewest number of cells (supplemental Table S2).

**Table V:** Number of strains analyzed, corresponding number of MS analyses and the resulting number of species-unique peptides found for each of the species

Species	Number of strains	Number of MS analyses	Number of species-unique peptides
*S. aureus*	12	36	5,847
*M. catarrhalis*	11	33	5,810
*H. influenzae*	9	26	2,978
*S. pneumoniae*	7	21	782

**Fig. 2.**
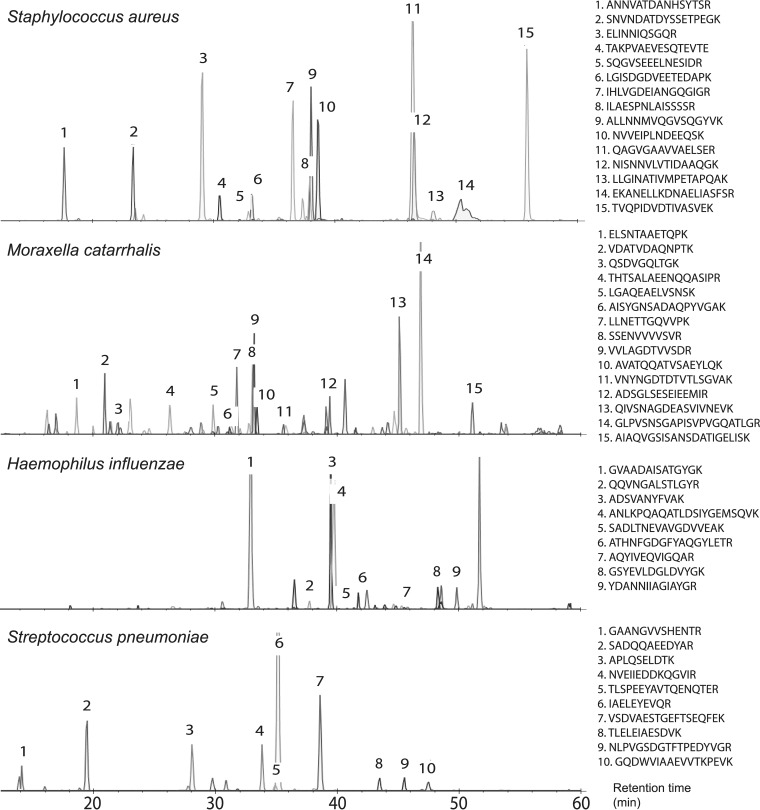
**Direct analyses of clinical respiratory tract samples, using PRM, targeting the most promising peptide biomarker candidates, presented in Tables I–IV.** The peptide intensities are summed up fragment ion intensities of the peptides′ most abundant charge state. Whenever peptides contain a methionine the more abundant oxidized form is shown in the spectra. The peptide peaks are labeled with numbers corresponding to their sequences.

Finally, as a proof-of-concept, a PRM method was developed, offering increased sensitivity and high selectivity (22), by targeting the most suitable peptide biomarker candidates identified in the verification phase (Tables I–IV, supplemental Data S5–S8, supplemental Table S4, Fig. 2). The peptide identities were verified by aligning the retention time together with correct transitions and mass accuracy.

## DISCUSSION

The selection of species-unique peptides was performed throughout the phases of discovery, qualification and verification (Fig. 1, supplemental Fig. S4). The purpose of these phases was to narrow down the number of species-unique peptides for determining the most suitable peptide biomarker candidates, from the starting point of analyses of bacterial reference cultures in the discovery phase, representing the genomic and proteomic variation of the species included in the study. In the qualification phase, the species-unique peptides detected in the lowest number of spiked cells displayed suitable properties for ionization, fragmentation and detection in the MS-analyses. Also, they were not suppressed by contaminating peptides of human origin from the clinical samples. Furthermore, the results show that sufficient amounts of bacterial cells were recovered during the removal of the human biomass, also suggesting that the limited amount of bacterial pathogen material in clinical respiratory tract samples can be recovered for detection in the MS-analysis.

The results from the subsequent verification phase demonstrate the importance of confirming data stemming from cultures of bacterial reference strains, by analyses of clinical samples. As expected, not all the species-unique peptides identified in the bacterial cultures were detected in the clinical samples. During traditional protocols including cultivation, the conditions are selected to best promote growth for recovery of enough biomass for downstream analyses. However, during invasion of the host, pathogens are known to experience stress, such as nutrient limitation, low pH, etc. Exposure to host environments also triggers virulence responses by pathogens and, thus, virulence factors may be expressed and present in high levels in clinical samples, whereas they may be present at limited levels in culture. Therefore, pathogens display different protein profiles *in vivo*, compared with what is observed in defined cultivation conditions (43, 44).

Differences in protein profiles for cultured bacteria and clinical samples can be seen clearly in the analyses of *S. pneumoniae* and *H. influenzae* (Table III and IV). For these two species, 4 of the most promising species-unique peptides of *H. influenzae*, and 13 for *S. pneumoniae*, identified in the clinical samples, were not found in the analyses of any of the cultured bacterial reference strains. For *M. catarrhalis* and *S. aureus*, many of the peptide biomarker candidates originated from highly abundant cytosolic proteins, including ribosomal proteins. Because cytosolic house-keeping proteins, in general, are present in relatively high levels, regardless of growth conditions, the most prominent peptide biomarker candidates would most likely originate from the house-keeping proteins when analyzing clinical samples. These results are consistent with traditional gene-based approaches and MALDI-TOF MS, which both commonly use house-keeping genes and proteins as targets for identification. In contrast, many of the proteins identified from the peptide biomarker candidates for *S. pneumoniae* and *H. influenzae* include those associated with the surface of the cells. This might be because of the differences in taxonomic structure of the different species. *M. catarrhalis* and *S. aureus* are phylogenetically more distant from their closest related species and as a result their house-keeping proteins, including ribosomal proteins, do not display substantial sequence homology of the species closest to them. However, for *S. pneumoniae* and *H. influenzae*, the taxonomic structures around these species are more complex and the house-keeping proteins, including ribosomal proteins, display a higher degree of sequence homology to closely related species. Therefore, it may be more difficult to find peptide biomarker candidates originating from their house-keeping proteins. Surface-associated proteins have different functions, helping the bacteria survive in diverse and dynamic ecological niches and, particularly, these proteins are often involved in host-pathogen interactions, effectively functioning, as virulence factors (18). Many of the proteins identified from *S. pneumoniae* and *H. influenzae* by their respective peptide biomarker candidates belong to the group of surface-associated virulence factors. This can be explained by the fact that these proteins are the ones differentiating them from their closest relatives, as well as being expressed significantly in clinical samples.

In conclusion, the aim of this study was to initially identify species-unique peptides in cultures of bacterial reference strains from respiratory tract infectious bacteria (*S. aureus*, *M. catarrhalis*, *H. influenzae* and *S. pneumoniae*) and subsequently determine the most promising and applicable peptide biomarker candidates in clinical samples. Previous proteomic studies, focused on discovery of peptide biomarker candidates for infectious disease diagnostics, have mostly been performed using *in vitro* model system samples, mainly because of analytical challenges such as recovery of sufficient amount of bacterial proteins from human clinical samples and the high background of human contaminating proteins obstructing the detection of peptide biomarkers from bacteria. In this study, a simple workflow was developed, including removal of human material from clinical respiratory tract samples, whereas still being able to recover sufficient amounts of bacteria for detection of peptide biomarker candidates. Importantly, several hundreds of clinical respiratory tract samples were analyzed directly, without any culturing, thus confirming the presence of peptide biomarker candidates in the clinical samples and, at the same time, their relevance for identifications of the pathogens and as diagnostic biomarkers.

In further studies, the peptide biomarker candidates, will be employed in the development of a targeted MS-approach, as demonstrated here (Fig. 2). Targeted approaches, such as PRM and SRM/MRM (Selected Reaction Monitoring/Multiple reaction monitoring) have several advantages, compared with discovery phase studies, such as higher sensitivity and specificity, simplified MS-analysis and data evaluation (22). In this study, MS proteomics analyses of clinical samples that were confirmed to be positive for a respiratory tract pathogen, determined by standard clinical microbiology methodologies, was employed as a cost-effective approach for identifying peptides from the relevant pathogens included in this study. Notably, samples were frozen until correct identifications could be confirmed by standard means, although, that freezing step may have had a negative effect on some species, sensitive to freezing, thus reducing the number of intact cells prior to the sample preparation for the MS analysis workflow. For comparison of the peptide biomarker approach *versus* traditional culture-based methods for clinical microbiology diagnostics, the experimental design would be different, *i.e.* samples would not be frozen prior to processing and more of the sample volume would be dedicated and processed for proteotyping. Furthermore, targeted MS approaches, such as parallel reaction monitoring (PRM) would be employed, as demonstrated here by the proof-of-concept experiment, shown in Fig. 2, wherein a small sub-set of positive clinical samples were analyzed, targeting only the peptide biomarker candidates presented in Tables I–IV. In the continued development of the targeted approach, a larger ensemble of peptide biomarkers (selected from the species-unique peptides in supplemental Table S2) could be employed. Further studies are necessary to compare the use of MS-based peptide biomarkers for identifying respiratory tract pathogens to traditional methodologies - including cultivation-dependent techniques such as MALDI-TOF MS - in terms of sensitivity, specificity, speed, and cost. However, as demonstrated here with the proof-of concept PRM analysis, we show the value of a targeted approach for future high throughput and specific detection of bacteria within complex samples, such as clinical respiratory tract samples, *i.e.* without prior cultivation steps.

## DATA AVAILABILITY

The mass spectrometry proteomics data (MSMS-spectra for all species-unique peptides presented in Tables I–IV, as well as raw-files and PD1.4 search files of representative clinical samples) have been deposited to the ProteomeXchange Consortium via the PRIDE partner repository with the data set identifiers PXD014522.

## Supplementary Material

supplemental Table S2

Supplemental File 1

Supplemental Table 1

Supplemental Figure 1

Supplemental Figure 2

Supplemental Figure 3

Supplemental Figure 4

Supplemental Figures 9-12

Supplemental Data 8

Supplemental Data 7

Supplemental Data 6

Supplemental Data 5

Supplemental Data 4

Supplemental Data 3

Supplemental Data 2

Supplemental Data 1

Supplemental Figures 5-8

Supplemental Table 2

Supplemental Table 3

Supplemental Table 4

## Footnotes

^1^ The abbreviations used are:

RTI: respiratory tract infection
AMR: antimicrobial resistance
TCUP: typing and characterization of bacteria using bottom-up tandem MS proteomics
HRAM: high-resolution accurate-mass
LPI: lipid-based protein immobilization
ANI: average nucleotide identity
BLAST: Basic Local Alignment Search Tool
SDC: sodium deoxycholate
NCBI: National Center for Biotechnology Information
PRM: parallel reaction monitoring
SRM: selected reaction monitoring
MRM: multiple reaction monitoring.
