# Supplementary material for: Discovery of Species-unique Peptide Biomarkers of Bacterial Pathogens by Tandem Mass Spectrometry-based Proteotyping
Source: Mol Cell Proteomics. 2020 Jan 15;19(3):518–28. doi: 10.1074/mcp.RA119.001667 (PMC7050107; doi:10.1074/mcp.RA119.001667)
Supplement: supplemental Table S2 [file RA119.001667_index.html]

Supplement to Discovery of species-unique peptide biomarkers of bacterial pathogens by tandem mass spectrometry-based proteotyping | Molecular & Cellular Proteomics

## Supplemental Data

- Supplemental File 1 - X!Tandem settings
- Supplemental Table 1 - Bacterial strains from the CCUG included in the study
- Supplemental Figure 1 - Discovery phase illustration
- Supplemental Figure 2 - Qualification phase illustration
- Supplemental Figure 3 - Verification phase illustration
- Supplemental Figure 4 - Illustration showing the selection of species-unique peptides
- Supplemental Figures 9-12 - Tandem mass spectra and ion series of selected prominent peptide biomarker candidates
- Supplemental Data 8 - PRM list of peptides for Streptococcus pneumoniae
- Supplemental Data 7 - PRM list of peptides for Haemophilus influenzae
- Supplemental Data 6 - PRM list of peptides for Moraxella catarrhalis
- Supplemental Data 5 - PRM list of peptides for Staphylococcus aureus
- Supplemental Data 4 - nanoLCMSMS inclusion list of peptides for Streptococcus pneumoniae
- Supplemental Data 3 - nanoLCMSMS inclusion list of peptides for Haemophilus influenzae
- Supplemental Data 2 - nanoLCMSMS inclusion list of peptides for Moraxella catarrhalis
- Supplemental Data 1 - nanoLCMSMS inclusion list of peptides for Staphylococcus aureus
- Supplemental Figures 5-8 - Supplemental Figures 5-8 showing the progressive increase in the number of species-unique peptides after adding successive MS data from triplicate analyses of different strains
- Supplemental Table 2 - All species-unique peptides identified by bacterial cultures.
- Supplemental Table 3 - Species unique peptide biomarkers identified in dilution-series samples.
- Supplemental Table 4 - All species-unique peptides presented in Tables 1-4.
