# Supplementary material for: Discovery of Species-unique Peptide Biomarkers of Bacterial Pathogens by Tandem Mass Spectrometry-based Proteotyping
Source: Mol Cell Proteomics. 2020 Jan 15;19(3):518–28. doi: 10.1074/mcp.RA119.001667 (PMC7050107; doi:10.1074/mcp.RA119.001667)
Supplement: Supplemental Table 1 [file 154211_2_supp_457756_q437ts.docx]

Supplemental Table 1. Bacterial strains selected from the Culture Collection University of Gothenburg (CCUG). The strains were selected to reflect the genomic diversity within each species.

| *Streptococcus pneumoniae* | *Haemophilus influenzae* | *Moraxella catarrhalis* | *Staphylococcus aureus* |
| --- | --- | --- | --- |
| 28588T | 23945T | 353T | 41582T |
| 35272 | 35273 | 34455 | 62707 |
| 1350 | 4559 | 63408 | 68900 |
| 11780 | 60440 | 18284 | 62271 |
| 33774 | 26214 | 56314 | 1964 |
| 7206 | 23969 | 36757 | 39740 |
| 35180 | 33775 | 41836 | 49245 |
|  | 32226 | 18283 | 64138 |
|  | 9188 | 27321 | 1979 |
|  |  | 33013 | 69160 |
|  |  | 63533 | 62274 |
|  |  |  | 1988 |
|  |  |  | 1914 |
