# Supplementary material for: Discovery of Species-unique Peptide Biomarkers of Bacterial Pathogens by Tandem Mass Spectrometry-based Proteotyping
Source: Mol Cell Proteomics. 2020 Jan 15;19(3):518–28. doi: 10.1074/mcp.RA119.001667 (PMC7050107; doi:10.1074/mcp.RA119.001667)
Supplement: Supplemental Figure 1 [file 154211_2_supp_457757_q437vs.docx]

Supplemental Figure 1. Workflow for generating a database of peptide biomarkers, identified by MS-proteotyping. Peptides detected in the largest number of MS runs are ranked highest, whereas peptides detected in the fewest number of MS runs are ranked lowest. The highest-ranked peptides are considered to be the most promising biomarker candidates.
