# Supplementary material for: Discovery of Species-unique Peptide Biomarkers of Bacterial Pathogens by Tandem Mass Spectrometry-based Proteotyping
Source: Mol Cell Proteomics. 2020 Jan 15;19(3):518–28. doi: 10.1074/mcp.RA119.001667 (PMC7050107; doi:10.1074/mcp.RA119.001667)
Supplement: Supplemental Figure 2 [file 154211_2_supp_457758_q4377s.docx]

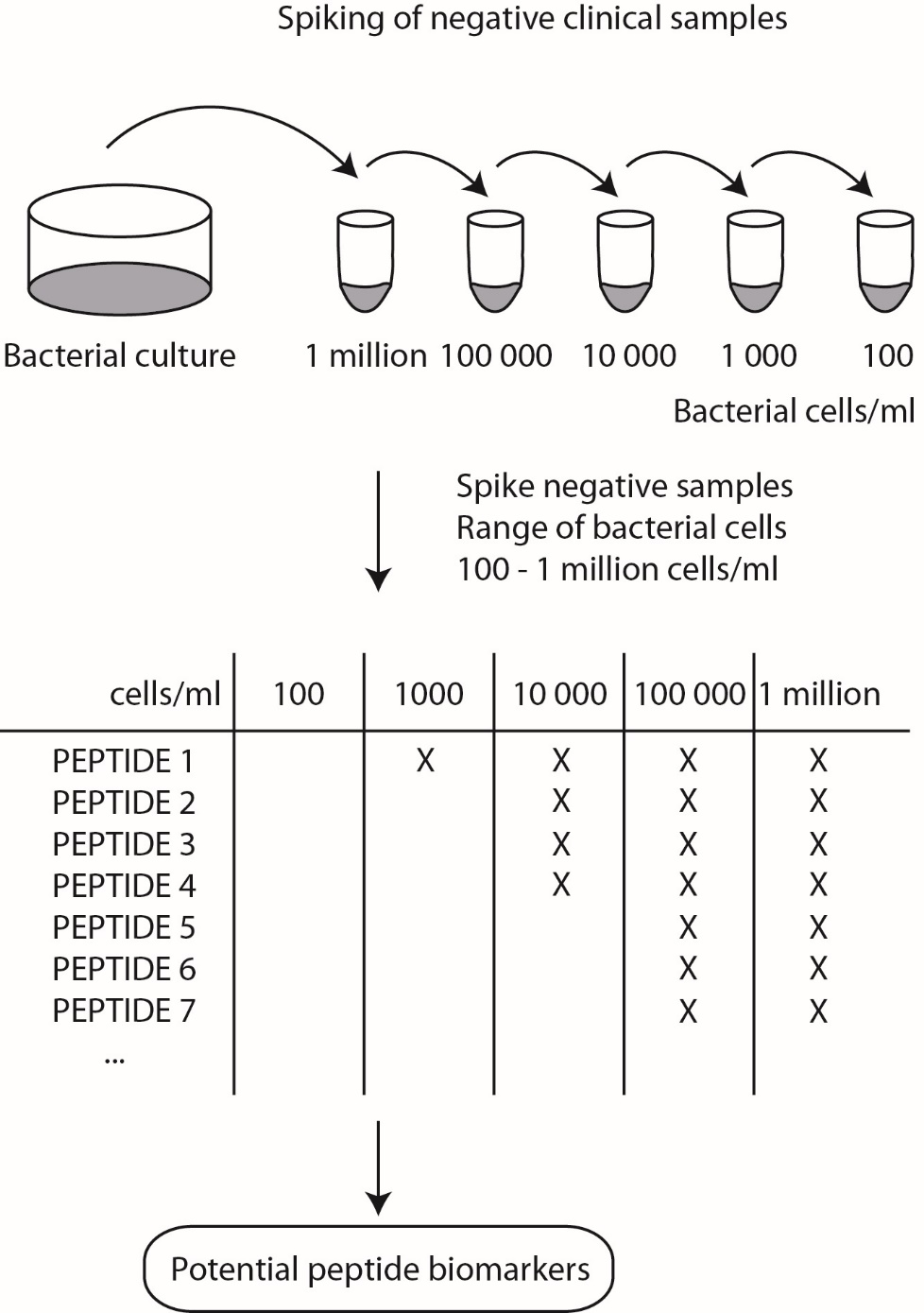
Supplemental Figure 2. Workflow for analyses of negative samples spiked with controlled numbers of cells and rankings of peptides, according to sensitivity of detection. The peptides found in the samples with fewest cells added displayed suitable properties for ionization, fragmentation and detection in the MS-analyses, and were thus deemed to be promising peptide biomarker candidates.
