# Supplementary material for: Discovery of Species-unique Peptide Biomarkers of Bacterial Pathogens by Tandem Mass Spectrometry-based Proteotyping
Source: Mol Cell Proteomics. 2020 Jan 15;19(3):518–28. doi: 10.1074/mcp.RA119.001667 (PMC7050107; doi:10.1074/mcp.RA119.001667)
Supplement: Supplemental Figure 3 [file 154211_2_supp_457759_q437cc.docx]

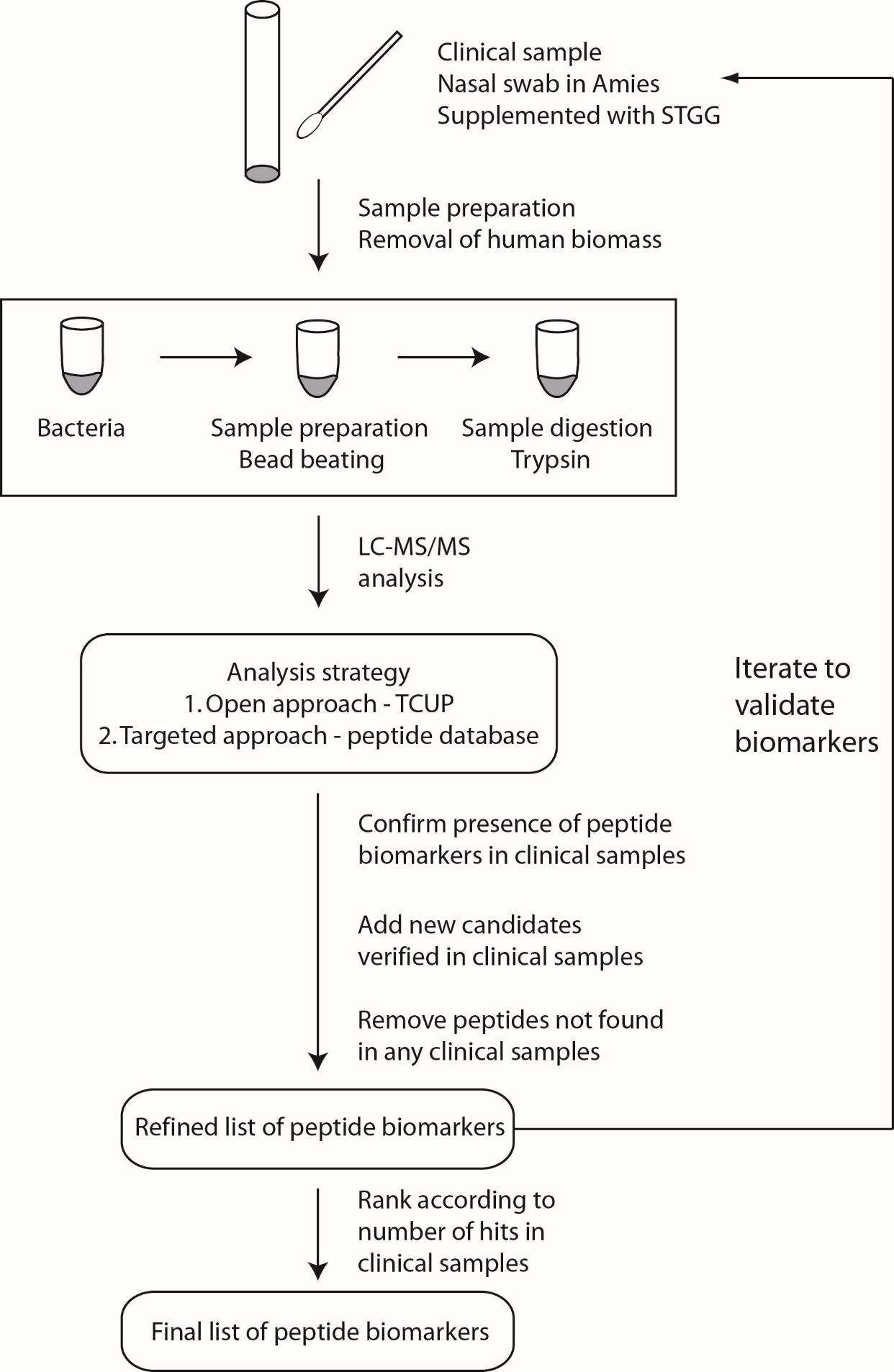


Supplemental Figure 3. Workflow for analyzing clinical samples, followed by bioinformatics processing, using open (TCUP) and targeted (peptide database) approaches, to identify peptide biomarkers by validating their detection and identification in clinical samples.
