# Supplementary material for: Discovery of Species-unique Peptide Biomarkers of Bacterial Pathogens by Tandem Mass Spectrometry-based Proteotyping
Source: Mol Cell Proteomics. 2020 Jan 15;19(3):518–28. doi: 10.1074/mcp.RA119.001667 (PMC7050107; doi:10.1074/mcp.RA119.001667)
Supplement: Supplemental Figure 4 [file 154211_2_supp_457760_q437nn.docx]

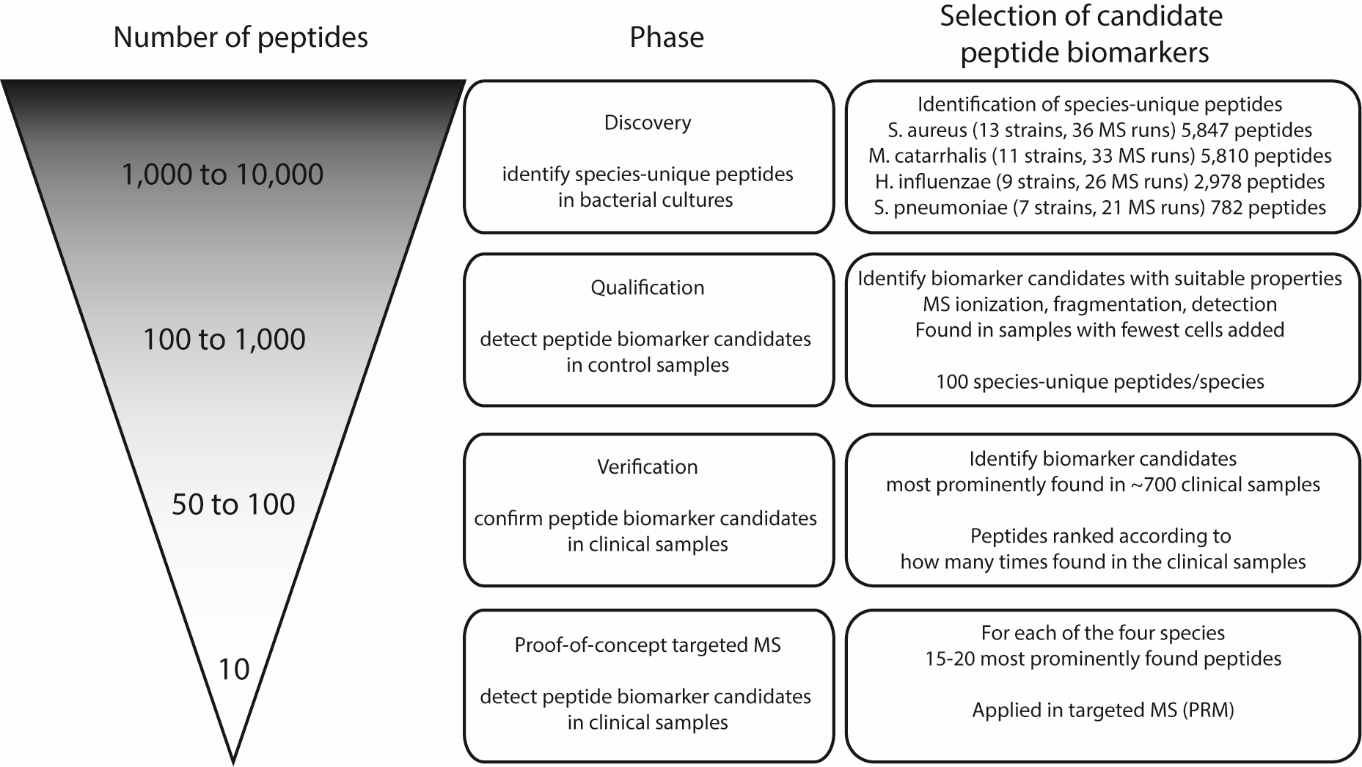


Supplemental Figure 4. Schematic illustration showing the selection of species-unique peptides throughout the phases of Discovery, Qualification and Verification.

In the Discovery phase, several strains from each of the four species were analyzed, in triplicates. The number of strains, MS runs and the number of species-unique peptides identified are shown. The species-unique peptides were ranked according to how many times they were found in the total number of MS runs. The top 100 ranked peptides were used to create two inclusion lists of 50 peptides in each inclusion list.

In the Qualification phase, clinical samples deemed negative by traditional culture-based methodologies, were selected from the clinical microbiology routine laboratory, and spiked with defined amounts of bacterial cells, from cultures of the reference bacterial type strains of each of the species included in the study. Bacterial cells were added in a range of 1 million cells/ml down to 100 cells/ml. Peptides found in the samples containing fewest amounts of added cells were deemed as promising peptide biomarker candidates. The listed peptides were re-ranked so that peptides detected in the samples with a lower number of spiked pathogenic bacteria were ranked higher. Again, two inclusion lists of 50 peptides in each list, were generated.

In the Verification phase, “positive control” samples, containing one or more of the four target bacterial species, were analyzed directly, without any culturing, in order to verify the presence of the peptide biomarker candidates in patient samples, where the protein expression might be different as compared to a plate culture. The clinical samples were analyzed in batches and the inclusion lists for the four target species were revised after the analysis of each batch, according to the following criteria: 1) Identified peptides from the list were verified as a peptide biomarker candidate by its presence in clinical samples; 2) Peptides identified in the clinical samples by TCUP, but not present in the inclusion lists, were added to updated versions of the inclusion lists; 3) Peptide biomarker candidates present in the initial inclusion lists, but not detected in the clinical samples, were removed from updated inclusion lists or were given a lower ranking (Supplemental Figure 3, Supplemental data 1-4). The peptides most prominently detected in clinical samples, and their corresponding proteins, as well as the number of times they were detected in the cultures of bacterial reference strains, are presented in Tables 1-4.
