# Supplementary material for: Discovery of Species-unique Peptide Biomarkers of Bacterial Pathogens by Tandem Mass Spectrometry-based Proteotyping
Source: Mol Cell Proteomics. 2020 Jan 15;19(3):518–28. doi: 10.1074/mcp.RA119.001667 (PMC7050107; doi:10.1074/mcp.RA119.001667)
Supplement: Supplemental Figures 9-12 [file 154211_2_supp_457761_q43755.docx]

Supplemental Figure 9. Tandem mass spectra and ion series for the most prominent *S. pneumoniae* peptide: VSDVAESTGEFTSEQFEK.


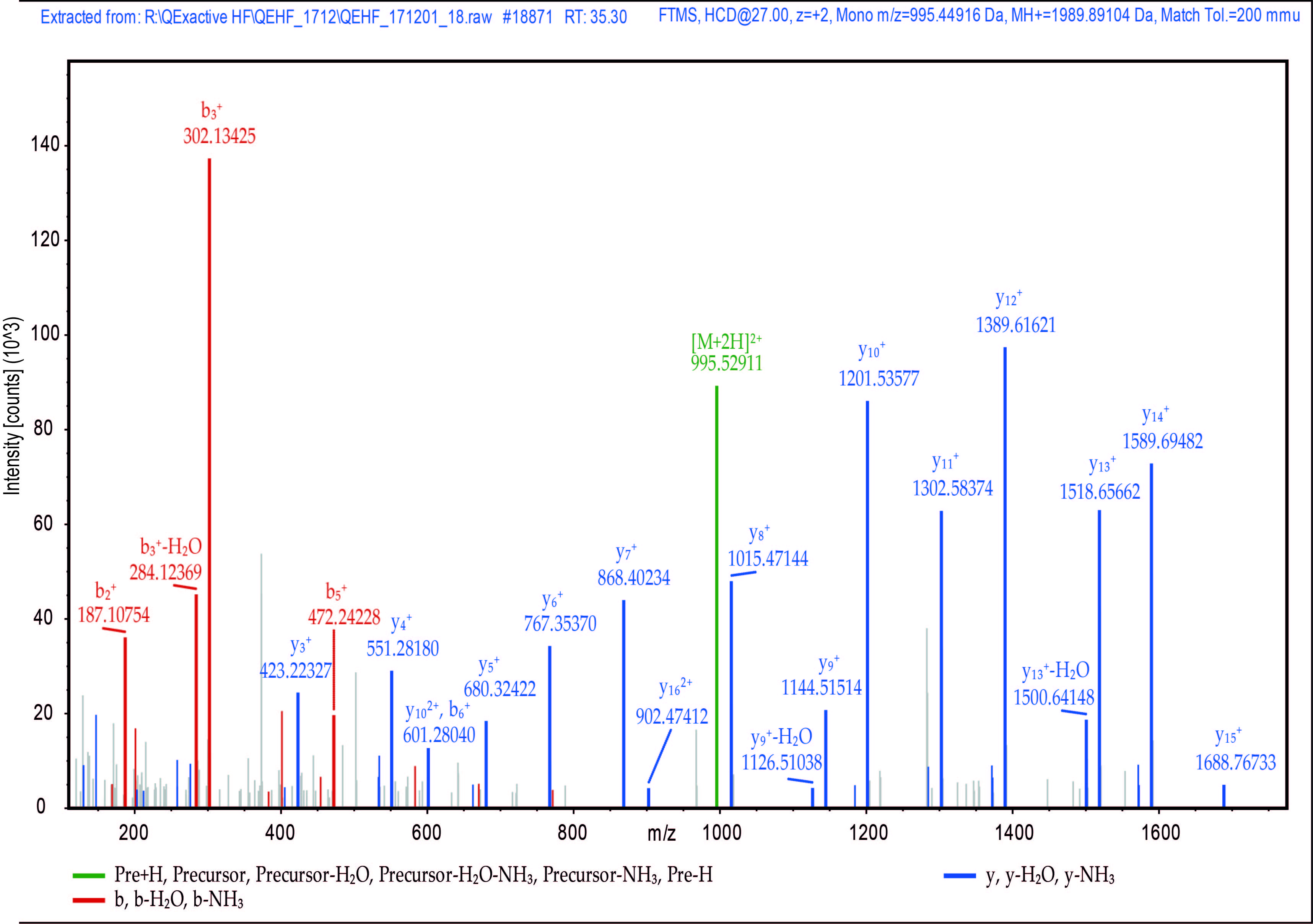


| **#1** | **b⁺** | **b²⁺** | **Seq.** | **y⁺** | **y²⁺** | **#2** |
| --- | --- | --- | --- | --- | --- | --- |
| 1 | 100.07570 | 50.54149 | V |  |  | 18 |
| 2 | 187.10773 | 94.05750 | S | 1890.82404 | 945.91566 | 17 |
| 3 | 302.13468 | 151.57098 | D | 1803.79201 | 902.39964 | 16 |
| 4 | 401.20310 | 201.10519 | V | 1688.76506 | 844.88617 | 15 |
| 5 | 472.24022 | 236.62375 | A | 1589.69664 | 795.35196 | 14 |
| 6 | 601.28282 | 301.14505 | E | 1518.65952 | 759.83340 | 13 |
| 7 | 688.31485 | 344.66106 | S | 1389.61692 | 695.31210 | 12 |
| 8 | 789.36253 | 395.18490 | T | 1302.58489 | 651.79608 | 11 |
| 9 | 846.38400 | 423.69564 | G | 1201.53721 | 601.27224 | 10 |
| 10 | 975.42660 | 488.21694 | E | 1144.51574 | 572.76151 | 9 |
| 11 | 1122.49502 | 561.75115 | F | 1015.47314 | 508.24021 | 8 |
| 12 | 1223.54270 | 612.27499 | T | 868.40472 | 434.70600 | 7 |
| 13 | 1310.57473 | 655.79100 | S | 767.35704 | 384.18216 | 6 |
| 14 | 1439.61733 | 720.31230 | E | 680.32501 | 340.66614 | 5 |
| 15 | 1567.67591 | 784.34159 | Q | 551.28241 | 276.14484 | 4 |
| 16 | 1714.74433 | 857.87580 | F | 423.22383 | 212.11555 | 3 |
| 17 | 1843.78693 | 922.39710 | E | 276.15541 | 138.58134 | 2 |
| 18 |  |  | K | 147.11281 | 74.06004 | 1 |

Supplemental Figure 10. Tandem mass spectra and ion series for the most prominent *H. influenzae* peptide: GVAADAISATGYGK.


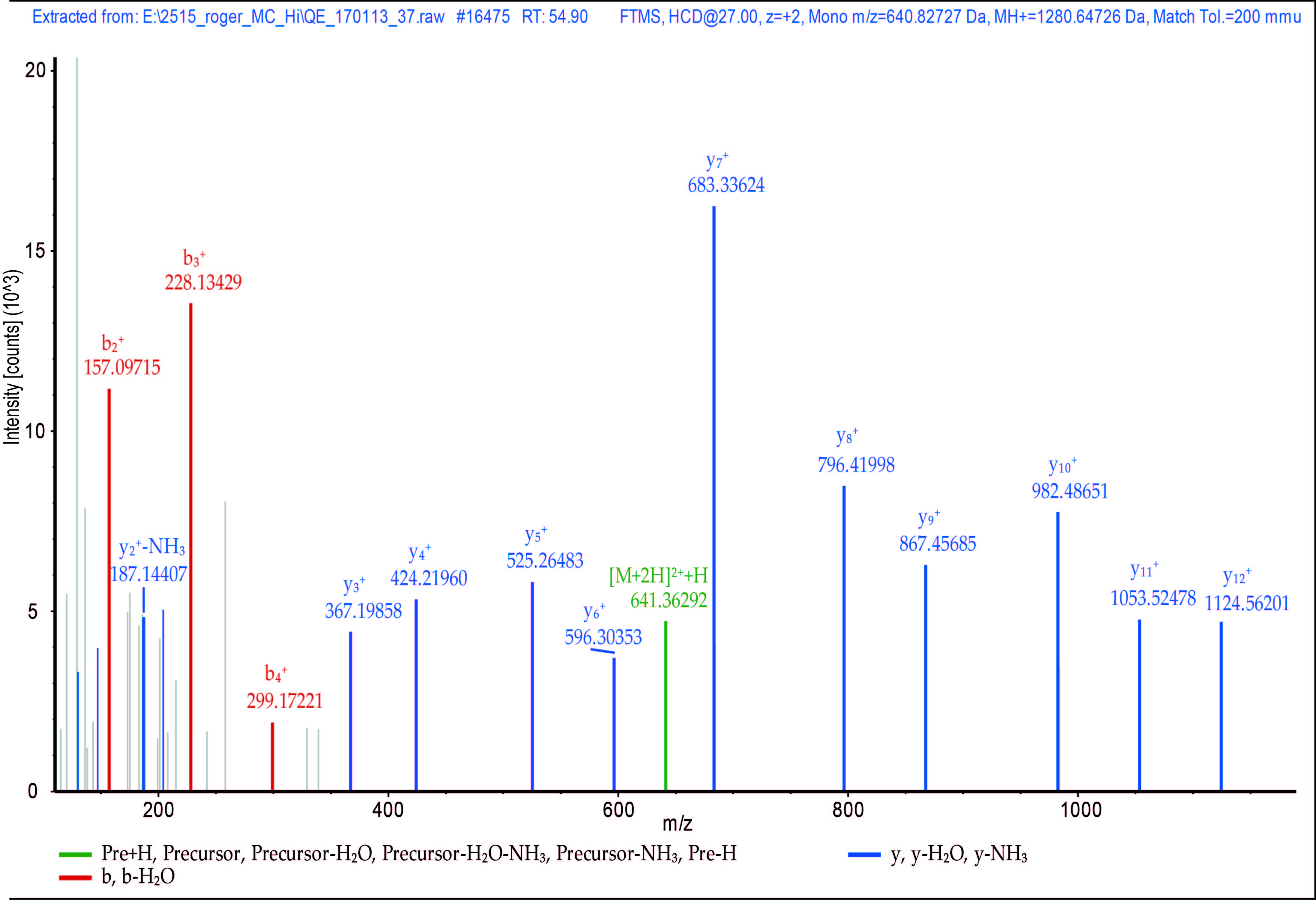


| **#1** | **Immonium** | **b⁺** | **b²⁺** | **Seq.** | **x⁺** | **x²⁺** | **y⁺** | **y²⁺** | **#2** |
| --- | --- | --- | --- | --- | --- | --- | --- | --- | --- |
| 1 | 30.03383 | 58.02875 | 29.51801 | G |  |  |  |  | 14 |
| 2 | 72.08078 | 157.09717 | 79.05222 | V | 1249.60597 | 625.30662 | 1223.62670 | 612.31699 | 13 |
| 3 | 44.04948 | 228.13429 | 114.57078 | A | 1150.53755 | 575.77241 | 1124.55828 | 562.78278 | 12 |
| 4 | 44.04948 | 299.17141 | 150.08934 | A | 1079.50043 | 540.25385 | 1053.52116 | 527.26422 | 11 |
| 5 | 88.03931 | 414.19836 | 207.60282 | D | 1008.46331 | 504.73529 | 982.48404 | 491.74566 | 10 |
| 6 | 44.04948 | 485.23548 | 243.12138 | A | 893.43636 | 447.22182 | 867.45709 | 434.23218 | 9 |
| 7 | 86.09643 | 598.31955 | 299.66341 | I | 822.39924 | 411.70326 | 796.41997 | 398.71362 | 8 |
| 8 | 60.04439 | 685.35158 | 343.17943 | S | 709.31517 | 355.16122 | 683.33590 | 342.17159 | 7 |
| 9 | 44.04948 | 756.38870 | 378.69799 | A | 622.28314 | 311.64521 | 596.30387 | 298.65557 | 6 |
| 10 | 74.06004 | 857.43638 | 429.22183 | T | 551.24602 | 276.12665 | 525.26675 | 263.13701 | 5 |
| 11 | 30.03383 | 914.45785 | 457.73256 | G | 450.19834 | 225.60281 | 424.21907 | 212.61317 | 4 |
| 12 | 136.07568 | 1077.52117 | 539.26422 | Y | 393.17687 | 197.09207 | 367.19760 | 184.10244 | 3 |
| 13 | 30.03383 | 1134.54264 | 567.77496 | G | 230.11355 | 115.56041 | 204.13428 | 102.57078 | 2 |
| 14 |  |  |  | K | 173.09208 | 87.04968 | 147.11281 | 74.06004 | 1 |

Supplemental Figure 11. Tandem mass spectra and ion series for the most prominent *M. catarrhalis* peptide: VDATVDAQNPTK.


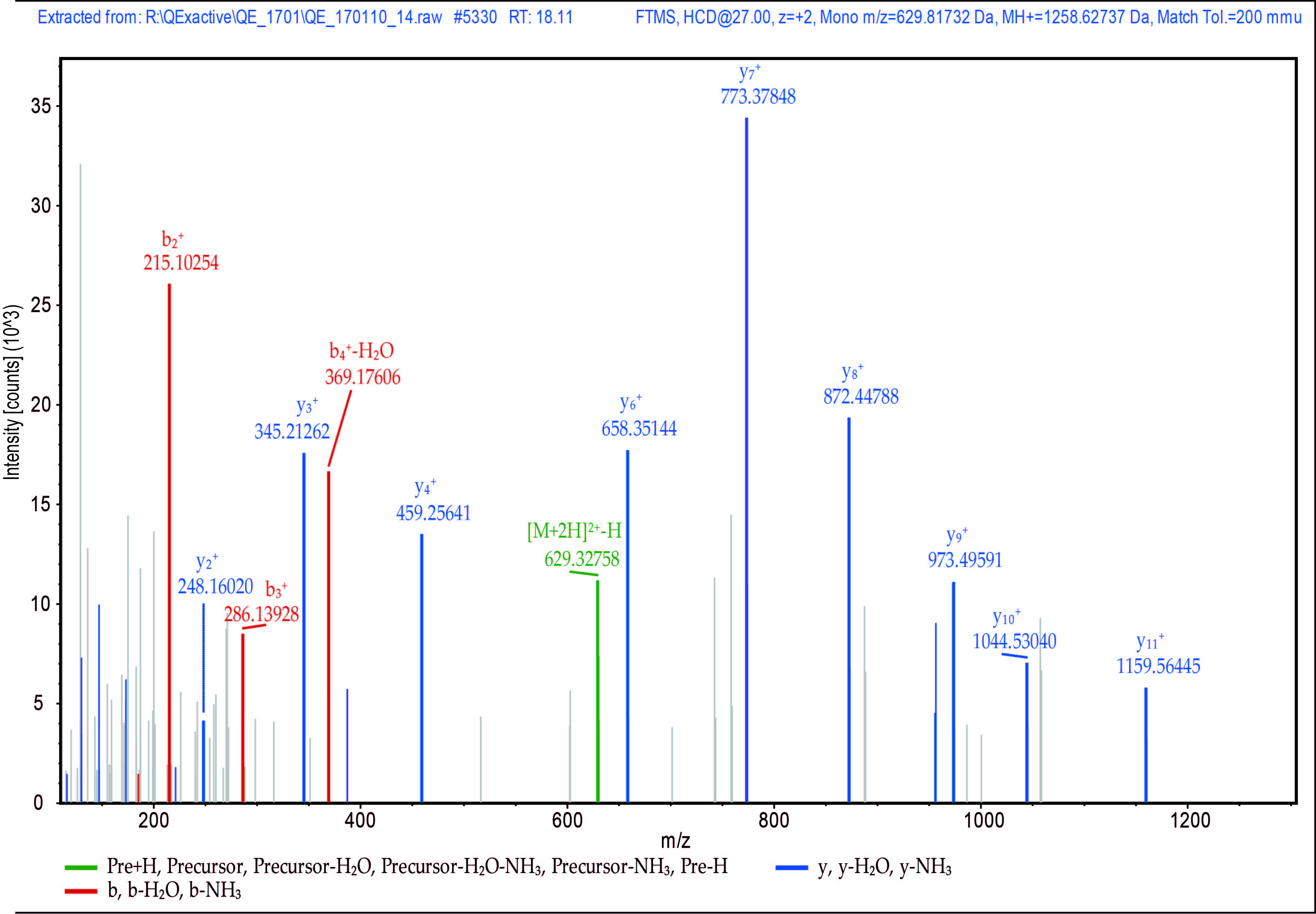


| **#1** | **b⁺** | **b²⁺** | **Seq.** | **y⁺** | **y²⁺** | **#2** |
| --- | --- | --- | --- | --- | --- | --- |
| 1 | 100.07570 | 50.54149 | V |  |  | 12 |
| 2 | 215.10265 | 108.05496 | D | 1159.55901 | 580.28314 | 11 |
| 3 | 286.13977 | 143.57352 | A | 1044.53206 | 522.76967 | 10 |
| 4 | 387.18745 | 194.09736 | T | 973.49494 | 487.25111 | 9 |
| 5 | 486.25587 | 243.63157 | V | 872.44726 | 436.72727 | 8 |
| 6 | 601.28282 | 301.14505 | D | 773.37884 | 387.19306 | 7 |
| 7 | 672.31994 | 336.66361 | A | 658.35189 | 329.67958 | 6 |
| 8 | 800.37852 | 400.69290 | Q | 587.31477 | 294.16102 | 5 |
| 9 | 914.42145 | 457.71436 | N | 459.25619 | 230.13173 | 4 |
| 10 | 1011.47422 | 506.24075 | P | 345.21326 | 173.11027 | 3 |
| 11 | 1112.52190 | 556.76459 | T | 248.16049 | 124.58388 | 2 |
| 12 |  |  | K | 147.11281 | 74.06004 | 1 |

Supplemental Figure 12. Tandem mass spectra and ion series for the most prominent *S. aureus* peptide: QAGVGAAVVAELSER.


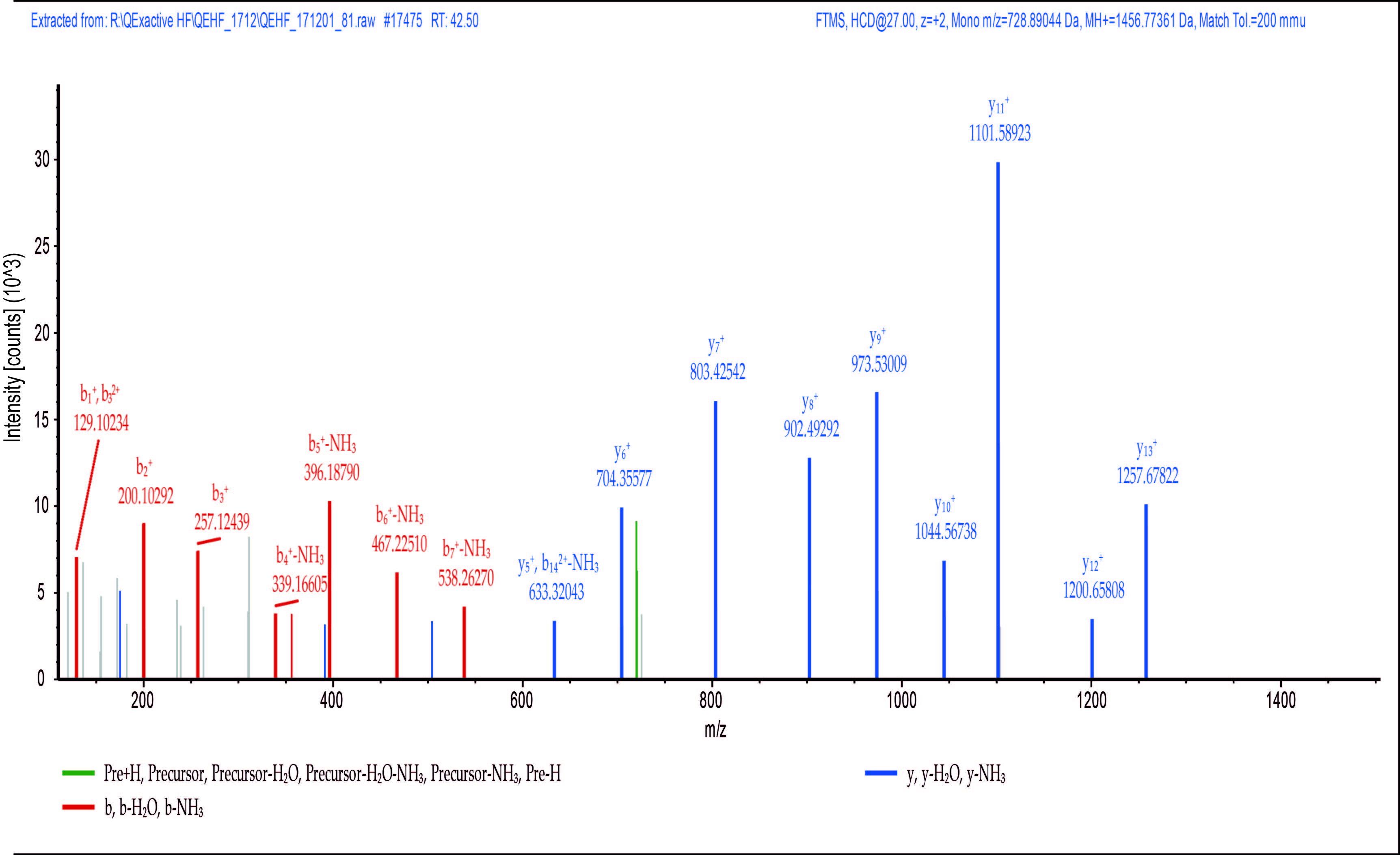


| **#1** | **b⁺** | **b²⁺** | **Seq.** | **y⁺** | **y²⁺** | **#2** |
| --- | --- | --- | --- | --- | --- | --- |
| 1 | 129.06586 | 65.03657 | Q |  |  | 15 |
| 2 | 200.10298 | 100.55513 | A | 1328.71694 | 664.86211 | 14 |
| 3 | 257.12445 | 129.06586 | G | 1257.67982 | 629.34355 | 13 |
| 4 | 356.19287 | 178.60007 | V | 1200.65835 | 600.83281 | 12 |
| 5 | 413.21434 | 207.11081 | G | 1101.58993 | 551.29860 | 11 |
| 6 | 484.25146 | 242.62937 | A | 1044.56846 | 522.78787 | 10 |
| 7 | 555.28858 | 278.14793 | A | 973.53134 | 487.26931 | 9 |
| 8 | 654.35700 | 327.68214 | V | 902.49422 | 451.75075 | 8 |
| 9 | 753.42542 | 377.21635 | V | 803.42580 | 402.21654 | 7 |
| 10 | 824.46254 | 412.73491 | A | 704.35738 | 352.68233 | 6 |
| 11 | 953.50514 | 477.25621 | E | 633.32026 | 317.16377 | 5 |
| 12 | 1066.58921 | 533.79824 | L | 504.27766 | 252.64247 | 4 |
| 13 | 1153.62124 | 577.31426 | S | 391.19359 | 196.10043 | 3 |
| 14 | 1282.66384 | 641.83556 | E | 304.16156 | 152.58442 | 2 |
| 15 |  |  | R | 175.11896 | 88.06312 | 1 |
