# Supplementary material for: Discovery of Species-unique Peptide Biomarkers of Bacterial Pathogens by Tandem Mass Spectrometry-based Proteotyping
Source: Mol Cell Proteomics. 2020 Jan 15;19(3):518–28. doi: 10.1074/mcp.RA119.001667 (PMC7050107; doi:10.1074/mcp.RA119.001667)
Supplement: Supplemental Figures 5-8 [file 154211_2_supp_457772_q437zv.docx]

Supplemental Figures 5-8 showing the progressive increase in the number of species-unique peptides after adding successive MS data from triplicate analyses of different strains - it was concluded that the number of strains selected per species was enough to find candidate species-unique peptide biomarkers.

Supplemental Figure 5. Number of species-unique peptides for *S. aureus* versus number of added strains (12 strains in triplicate MS analyses). The Y-axis shows the number of species-unique peptides, which is increased by additional MS runs (the number of MS runs is indicated on the X-axis).

Supplemental Figure 6. Number of species-unique peptides for *M. catarrhalis* versus number of added strains (11 strains in triplicate MS analyses). The Y-axis shows the number of species-unique peptides, which is increased by additional MS runs (the number of MS runs is indicated on the X-axis).

Supplemental Figure 7. Number of species-unique peptides for *H. influenzae* versus number of added strains (9 strains in triplicate MS analyses). The Y-axis shows the number of species-unique peptides, which is increased by additional MS runs (the number of MS runs is indicated on the X-axis).

Supplemental Figure 8. Number of species-unique peptides for *S. pneumoniae* versus number of added strains (7 strains in triplicate MS analyses). The Y-axis shows the number of species-unique peptides, which is increased by additional MS runs (the number of MS runs is indicated on the X-axis).
