# Supplementary material for: Discovery of Species-unique Peptide Biomarkers of Bacterial Pathogens by Tandem Mass Spectrometry-based Proteotyping
Source: Mol Cell Proteomics. 2020 Jan 15;19(3):518–28. doi: 10.1074/mcp.RA119.001667 (PMC7050107; doi:10.1074/mcp.RA119.001667)
Supplement: Supplemental Table 3 [file 154211_2_supp_457774_q437ww.docx]

Supplemental Table 3. Species unique peptide biomarkers identified in dilution-series samples containing 10^4^ cells/ml. For *S. aureus*, peptides were identified in dilution-series samples containing 10^3^ added cells/ml.

| *S. aureus (10^3^)* | *M. catarrhalis* | *H. influenzae* | *S. pneumoniae* |
| --- | --- | --- | --- |
| ITYTMIGDPSQTITR | VGDEIEIIGIKPTAK | GVAADAISATGYGK | VQYEGGTEDELIR |
| AILNNENNVLNVSIQLDGQYGGHK | TDEQLQAELDNK | AVVYNNEGTNVELGGR | VSDVAESTGEFTSEQFEK |
| GLEVGQIVESGAEADIK | GLITNSIENTNNITK | FGQGEAPVVAAPEVVSK | GLDVTDEEGDDVTNGIFVGAK |
| TVEVDGYNAIQVGFEDK | QIVSNAGDEASVIVNEVK | DGQVTGALATLGEPYK | SQTEQGEINIER |
| SINPADTSQVIANASK | NTIEGENSVAIGSNNTVK | LSVIAEQSNSTR | PAPAPQPAPAPKPEK |
| GGLTDTFTNAFSSGNNVTQGVSVEVGEK | QQTEAIDALNK | YDANNIIAGIAYGR | AEADKPETEAGKER |
| NFDVLDEATGLAQR |  |  | GAANGVVSHENTR |
|  |  |  | AEGVATASETAEAASAAKPEEK |
